# Supplementary material for: Subjective Well-Being, Health and Socio-Demographic Factors Related to COVID-19 Vaccination: A Repeated Cross-Sectional Sample Survey Study from 2021–2022 in Urban Pakistan
Source: Int J Environ Res Public Health. 2023 Aug 8;20(16):6545. doi: 10.3390/ijerph20166545 (PMC10454671; doi:10.3390/ijerph20166545)
Supplement: Supplementary file 1 [file ijerph-20-06545-s001.zip › ijerph-2464933-supplementary.pdf]

Supplementary Material:

Table S1. The weighting scheme.

**Census 2017 [18] (Last Available Census)**			**Pooled Survey, 2021 & 2022**	
**City**	**Total Population**	**Sample Population**	**pweights [$(TP)_j$ /$(SP)_j$]**	**pweights-normalized [$(pw)_j$ /$\Sigma(pw)_j$]**
**(j)**	**$(TP)_j$**	**$(SP)_j$**	**$(pw)_j$**	**$(pw)^*_j$**
Lahore	11,126,285	1,125	9890.03111	0.16
Faisalabad	3,204,726	450	7121.61333	0.12
Rawalpindi	2,098,231	338	6207.78402	0.10
Multan	1,871,843	225	8319.30222	0.13
Islamabad	1,009,832	112	9016.35714	0.15
Karachi	14,916,456	1800	8286.92	0.13
Peshawar	1,970,042	270	7296.45185	0.12
Quetta	1,001,205	180	5562.25	0.09
Σ	-	-	61700.70967	1

Note: The last column reports the corresponding weights for the sample households.
